# Three Cases Showing the Use of Oil of Turpentine in the Treatment of Atrophy of the Shoulder Muscles1Read before the Fifteenth Annual Meeting of the Iowa State Veterinary Medical Association, Grand Rapids, Iowa, January 14 and 15, 1903.

**Published:** 1903-03

**Authors:** W. A. Heck


### THREE CASES SHOWING THE USE OF OIL OF TURPENTINE IN THE TREATMENT OF ATROPHY OF THE SHOULDER MUSCLES.[1]

By W. A. Heck, V.S.

Case I. Small bay mare, six years old, was injured in May, 1900, by pulling a heavy load of corn up a steep hill. The right scapular muscles began to shrink soon after and there was decided lameness. Oil of turpentine was injected at intervals of three inches into the scapular muscles and the animal turned to pasture. Three weeks later there was not much improvement and the injections repeated. Six weeks later no improvement was visible. It was decided to wait a little longer and allow the mare to run in pasture. Early in September she was caught up and the shoulder seemed to be a trifle improved, which indicated to me that nature was restoring the muscles without artificial aid. The turpentine injections were repeated. Next day the animal was unable to go out of the barn owing to stiffness and swelling of the scapular

[1] Read before the Fifteenth Annual Meeting of the Iowa State Veterinary Medical Association, Grand Rapids, Iowa, January 14 and 15, 1903.

muscles, the swelling extending down the limb to the knees. In three days the soreness and swelling disappeared sufficiently for the mare to be turned into a paddock, and she was put to light driving in a week: was used to buggy daily thereafter, and the swelling never went out of the shoulder enough to lead anyone to suspect that the animal had ever been "sweeneyed." The injections were made by simply putting a twitch on the nose and using no antiseptic precautions whatever, except to have the hypodermic syringe and needle afterwards sterilized. At last injection a small abscess formed, and when opened about five drachms of pus escaped. No treatment was given the abscess and the wound healed in a few days.

Case II. Young, black mare, weight 1000 pounds, was brought in fifteen miles to be treated for atrophy of both shoulders. The mare was "sweeneyed" plowing for corn. Both shoulders were injected at close intervals, using half a drachm in each side. Instructed the owner to take her home and turn her into pasture, and informed him that the next day the animal would be swollen some, but that we would have no good results if we got no swelling and cautioned him not to get frightened. Here let me say that it is always necessary to explain to the owner the conditions we may expect in a day or so after the injections. I advised the return of the animal in three weeks for a repetition of the treatment. According to promise, the animal was returned in three weeks, and to my agreeable surprise no treatment was necessary, as the muscles had so nearly filled that I thought there was no need of further treatment. This was a bad case, one of the worst I have ever seen, and the injury had been done only a couple of weeks. No antiseptic precautions were taken, and no abscesses resulted. The client was so well pleased that the following year he brought two more horses to be treated for scapular atrophy.

Case III. The third subject was a fine, young, bay express horse, purchased for shipment to Chicago market, suffering badly at the time of purchase from scapular atrophy, but, being such a nice horse, the buyer bought him and turned him over to me for treatment. I injected both shoulders, after washing the parts well with a strong solution of creogen, using about half a drachm of oil of turpentine in each shoulder. In about three weeks the shoulder was very much improved, in fact it was nearly well. The owner said to me, "He needs only a small dose just to fill the parts, and before the swelling goes out I will ship him." Accordingly, I sterilized my instruments, scrubbed the shoulders well with creogen solution and injected about one-half the former dose. The horse resisted a good deal, and after the injections seemed in great agony, lay down in the stall and kicked and rolled and in a few minutes was covered with perspiration. The shoulder swelled fearfully. In four days abscesses appeared at each point of injection; I think not a single point escaped. These abscesses were opened and syringed daily, and healed in a few days, but the muscles did not fill as satisfactorily as with the former injection. Six weeks have elapsed and still the shoulders are somewhat shrunken, and the owner is afraid to repeat the treatment, and I, myself, am nearly so, and he is such a fine horse I hesitate to apply a counter-irritant to the parts.

These three cases illustrate the success I have had with this therapeutic agent in these troubles, and, until I find a better treatment, I shall continue to use it.

---

Blood for Horse Food. During the past year the military authorities at Strassburg have been feeding a new food product to the horses of regiments stationed there, the experiment having resulted satisfactorily. This food consists principally of steam-dried, sterilized blood from the slaughter-houses, and is mixed with grain hulls, the husks of peanut kernels and molasses, and is sold in bags of 165 pounds at $2.75 per bag at retail. At the present time the demand for this dried blood-meal preparation is far greater than the daily capacity of the factory, the blood of the 300 cattle slaughtered every day in Strassburg not being sufficient; and if the German Government should adopt this food for all army horses, as seems not improbable, the company will be confronted with the necessity of establishing many new factories. In introducing this new feed it is advisable that the change be made gradually, until about half the old quantity has been cut off and replaced with from three to six pounds of the blood meal.

---

The following extract in regard to the work of the State Veterinarian of Pennsylvania show how it has impressed the Governor, Hon. Wm. A. Stone, and the Secretary of Agriculture, Prof. John Hamilton:

I desire to call attention particularly to the valuable investigations and discoveries made by the Veterinary Division, whose efforts to stamp out infectious diseases among live-stock have been most extensive and successful.—[From the Annual Message of the Governor to the Legislature, January 6, 1903.]

